# Chronic prostatitis alters the prostatic microenvironment and accelerates preneoplastic lesions in C57BL/6 mice

**DOI:** 10.1186/s40659-019-0237-4

**Published:** 2019-05-14

**Authors:** Yong Gao, Lijuan Wei, Chenbang Wang, Yuanjie Huang, Weidong Li, Tianyu Li, Chaohua Mo, Huali Qin, Xiaoge Zhong, Yun Wang, Aihua Tan, Zengnan Mo, Yonghua Jiang, Yanling Hu

**Affiliations:** 1 Department of Clinical Laboratory, The First Affiliated Hospital of Guangxi Medical University, Nanning, 530021 Guangxi China; 2 Life Sciences Institute, Guangxi Medical University, Nanning, 530021 Guangxi China; 3 Institute of Urology and Nephrology, First Affiliated Hospital of Guangxi Medical University, Nanning, 530021 Guangxi China; 4 Department of Chemotherapy, The Affiliated Tumor Hospital of Guangxi Medical University, Nanning, 530021 Guangxi China; 5 Center for Genomic and Personalized Medicine, Guangxi Medical University, Nanning, 530021 Guangxi China; 6 Guangxi Key Laboratory of Genomic and Personalized Medicine, Nanning, 530021 Guangxi China; 7 Guangxi Collaborative Innovation Center for Genomic and Personalized Medicine, Nanning, 530021 Guangxi China

**Keywords:** Chronic prostatitis, Prostatitis mouse model, Proliferative inflammatory atrophy, Prostatic intraepithelial neoplasia, Somatic genome mutation

## Abstract

**Background:**

Chronic prostatitis has been supposed to be associated with preneoplastic lesions and cancer development. The objective of this study was to examine how chronic inflammation results in a prostatic microenvironment and gene mutation in C57BL/6 mice.

**Methods:**

Immune and bacterial prostatitis mouse models were created through abdominal subcutaneous injection of rat prostate extract protein immunization (EAP group) or transurethral instillation of uropathogenic *E. coli* 1677 (*E. coli* group). Prostate histology, serum cytokine level, and genome-wide exome (GWE) sequences were examined 1, 3, and 6 months after immunization or injection.

**Result:**

In the EAP and *E. coli* groups, immune cell infiltrations were observed in the first and last months of the entire experiment. After 3 months, obvious proliferative inflammatory atrophy (PIA) and prostatic intraepithelial neoplasia (PIN) were observed accompanied with fibrosis hyperplasia in stroma. The decrease in basal cells (Cytokeratin (CK) 5+/p63+) and the accumulation of luminal epithelial cells (CK8+) in the PIA or PIN area indicated that the basal cells were damaged or transformed into different luminal cells. Hic1, Zfp148, and Mfge8 gene mutations were detected in chronic prostatitis somatic cells.

**Conclusion:**

Chronic prostatitis induced by prostate extract protein immunization or *E. coli* infection caused a reactive prostatic inflammation microenvironment and resulted in tissue damage, aberrant atrophy, hyperplasia, and somatic genome mutation.

**Electronic supplementary material:**

The online version of this article (10.1186/s40659-019-0237-4) contains supplementary material, which is available to authorized users.

## Introduction

Prostate cancer is one of the most common cancers and the leading cause of cancer in the male reproductive system worldwide. The International Agency for Research on Cancer reported that prostate cancer is the second most frequent cancer among males in 2018 [1]. Effective treatments for prostate cancer include radical prostatectomy, radiotherapy, and androgen deprivation therapy. However, about 15% of patients experience local recurrence or develop metastatic tumors [2]. If prostate cancer develops into castration-resistant prostate cancer, the treatment will be limited [3, 4]. Therefore, determining the key biological factors that lead to prostate cancer is important in establishing preventive treatments to halt the development of this cancer.

Prostatitis is a painful condition that involves the inflammation of the prostate and sometimes the areas around the prostate. Prostatitis can be divided into four types according to the prostatitis diagnosis guideline, and these types are chronic prostatitis/chronic pelvic pain syndrome, acute bacterial prostatitis, chronic bacterial prostatitis, and asymptomatic inflammatory prostatitis [5]. Prostatitis is one of the most common prostate conditions in young and middle-aged men. The estimated prevalence of medically diagnosed prostatitis is 9%, and the overall lifetime prevalence of prostatitis is estimated to be 14% [6]. The prevalence of NIH-IV prostatitis is 21.1% among 1868 asymptomatic men aged 19–78 years and increases with age [7]. Nearly 20% of human cancers are associated with chronic inflammation [8]. Inflammation is closely related to prostate cancer initiation and progression [9]. Innate or adaptive immune cells or inflammatory cytokines can promote tumor initiation and progression. However, the inflammation in the pathogenesis of preneoplastic prostate lesions remains unclear.

In this study, we set up two mouse models for prostatitis induced by prostate extract protein immunization (EAP group) and *E. coli* infection (*E. coli* group). The characteristics of the inflammatory responses and proliferating prostate epithelial cells were evaluated, and the impact of chronic inflammation on the development of chronic pelvic pain was explored. In addition, gene mutation in the prostate was identified. This study helps us understand the role of prostate inflammation in the initiation and progression of prostate cancer.

## Materials and methods

### Animals

Experiments were performed on C57BL/6 mice that were approximately 9–11 weeks old and weighed 25–30 g (Chang Zhou Cavens Laboratory Animal Ltd., Nanjing, China). All animal procedures were approved and performed under the guidance of the Institutional Animal Care and Use Committee of Guangxi Medical University. The study was approved by Ethics and Animal Subject Committee of Guangxi Medical University.

### Induction of prostatitis

For immune prostatitis, C57BL/6 male mice (9–11 weeks old) were injected subcutaneously in the abdominal flank with 200 μL of an emulsion consisting of equal amounts of water and complete Freund’s adjuvant (CFA, Sigma Aldrich, USA) with 100 μg of prostate extract protein (EAP group). Control mice were immunized with CFA alone (CFA group).

For bacterial prostatitis, C57BL/6 male mice (9–11 weeks old) were anaesthetized (pentobarbital sodium, Merck, Germany) and intraurethrally instilled with 200 μL of *E. coli* solution (2 × 10^6^ cfu) (*E. coli* group). Control mice were instilled with sterility phosphate buffer saline (PBS) (PBS group).

For the naïve group, no special treatment was included (naïve group).

### Behavioral testing for the assessment of pain thresholds

At different time points of 1, 3, and 6 months after immunization or infection, the mice were tested for tactile allodynia and referred hyperalgesia by applying von Frey filaments to the pelvic region. All mice were tested in individual chambers with a stainless-steel wire grid bottom. Eight forces from 0.008 to 4 g were applied with different sizes of von Frey filaments. Positive behavioral responses included sharp retraction of the abdomen, instant licking, scratching, and jumping. The withdrawal response frequency was determined for each filament as the percentage of positive responses to 10 stimuli, each of which was applied for 3 s with a 5 s interval between stimuli. Data were expressed as the mean percentage of the response frequency for each filament.

### ELISA

The serum level of C-reactive protein (CRP) was assessed as a nonspecific marker of inflammation and oxidative stress. CRP was measured using a Mouse C-Reactive Protein Quantizing ELISA kit (R&D Systems, USA). High concentrations of cytokine IL-17 have been shown to promote prostate cancer progression in mice. IL-17 was measured by using a Mouse IL-17 Quantizing ELISA kit (R&D Systems, USA). Absorbance was measured at 450 nm by using Varioskan Flash (Thermo Fisher Scientific, USA).

### Histology and Masson’s trichrome staining

The organs in the reproductive system, including prostate glands, testis, epididymis, and foreskin glands, were dissected. All organs were fixed in 10% buffered formalin overnight at 4 °C and embedded in paraffin. Sections (5 μm thick) were examined histologically through haematoxylin and eosin (H&E) staining. Masson’s trichrome staining was performed on prostate glands according to the Masson Stain Kit (Nanjing Jiancheng Bioengineering Institute, Nanjing, China).

### Immunohistochemistry

Sections were deparaffinized, and antigen retrieval was performed by steam heating in 0.01 M sodium citrate buffer (pH 6.0) for 10 min in a steamer. Cooled slides were incubated with 5% BSA blocking solution (Solarbio, Beijing, China) and incubated with primary antibodies overnight at 4 °C. The primary antibodies included mouse anti-K5 (ab190083, Abcam, UK), rabbit anti-K8 (ab59400, Abcam, UK), rabbit anti-CD45 (ab10558, Abcam, UK), rabbit anti-Hsp60 (ab45134, Abcam, UK), and rabbit anti-p63 (ab735, Abcam, UK). For fluorescence visualization, the slides were incubated with secondary antibodies (diluted 1:300 in PBS containing 0.05% Tween 20) and anti-rabbit lgG (H + L) Fragment (Alexa Fluor 555 Conjugate) (Cell Signaling Technology, USA). Sections were counterstained with DAPI (Sigma Aldrich, USA). Immunofluorescence staining was imaged with a fluorescence microscope (Olympus BX53, Olympus Optical Co., Ltd.).

### Transmission electron microscopy

The prostate dorsal lobe was cut into cubes of about 1 mm^3^, fixed with 3% glutaraldehyde (ALFA AESAR, USA) in 0.1 mol/L PBS buffer (pH 7.4) at 4 °C overnight, washed with PBS buffer three times, and post-fixed in 1% osmium tetroxide for 1 h at room temperature. Afterward, the cubes were washed with PBS buffer and dehydrated in ethanol with an increasing concentration (50%, 70%, 90%, and 100%) in a sequential manner, followed by dehydration in a mixture of 90% ethanol and 90% acetone and then with 100% acetone three times for 10 min each. The tissue cubes were embedded in Spurr resin (SPI-CHEM, USA) and cut into thin slices (70–100 nm thickness) by using a microtome (Leica, EM UC7, USA). Samples were collected on 200-mesh copper grids and double stained with 1% aqueous uranyl acetate and 1% lead citrate aqueous solution (TED PELLA, USA). Then, the samples were examined under an H-7650 transmission electron microscope (HITACHI, Japan).

### DNA preparation and exome sequencing

Genome-wide exome (GWE) sequencing was performed at Annoroad Gene Technology. Briefly, 0.6 μg of genomic DNA per sample was used as the input material for DNA sample preparation. Sequencing libraries were generated with the Agilent SureSelect Human All Exon kit (Agilent Technologies, CA, USA) following the manufacturer’s recommendations. Index codes were added to each sample. Complexes of library oligos and DNA fragments were captured, purified, and enriched via polymerase chain reaction (PCR). The DNA libraries were sequenced on an Illumina Hiseq platform, and 150 bp paired-end reads were generated. Valid sequencing data were mapped to mm10 (https://www.sanger.ac.uk/science/data/mouse-genomes-project) by using the Bwa (bwa) + SAMtools (samtools) + RSeQC [10] to obtain the original mapping results stored in a binary alignment map (BAM) format.

### GWE sequence analysis work flow

GATK (GenomeAnalysisTK.jar) were used to perform variant calling and identify SNP and InDels. Functional annotation is crucial because the link between genetic variations and diseases is clarified in this step. ANNOVAR software was applied to perform annotation on the variant call format (VCF) obtained previously. mm10 (refGene file) were used to characterize the detected variants. Annotation content contained the variant position, variant type, heatmaps depicting the total variant numbers per strain and the sum of strain-specific variants per two strain combinations were created in R by using [ggplot2] and [gridExtra] [11].

### Statistical analysis

All experiments were performed independently using six mice. Data were presented as mean ± standard error of the mean (SEM). Two-way analysis of variance and Student’s t test were used to determine the significance between groups. For all statistical tests, the 0.05 level of confidence was accepted for statistical significance.

## Result

### Immune injection or bacterial infection results in inflammation and immune reaction in the prostate

**Fig. 1:**
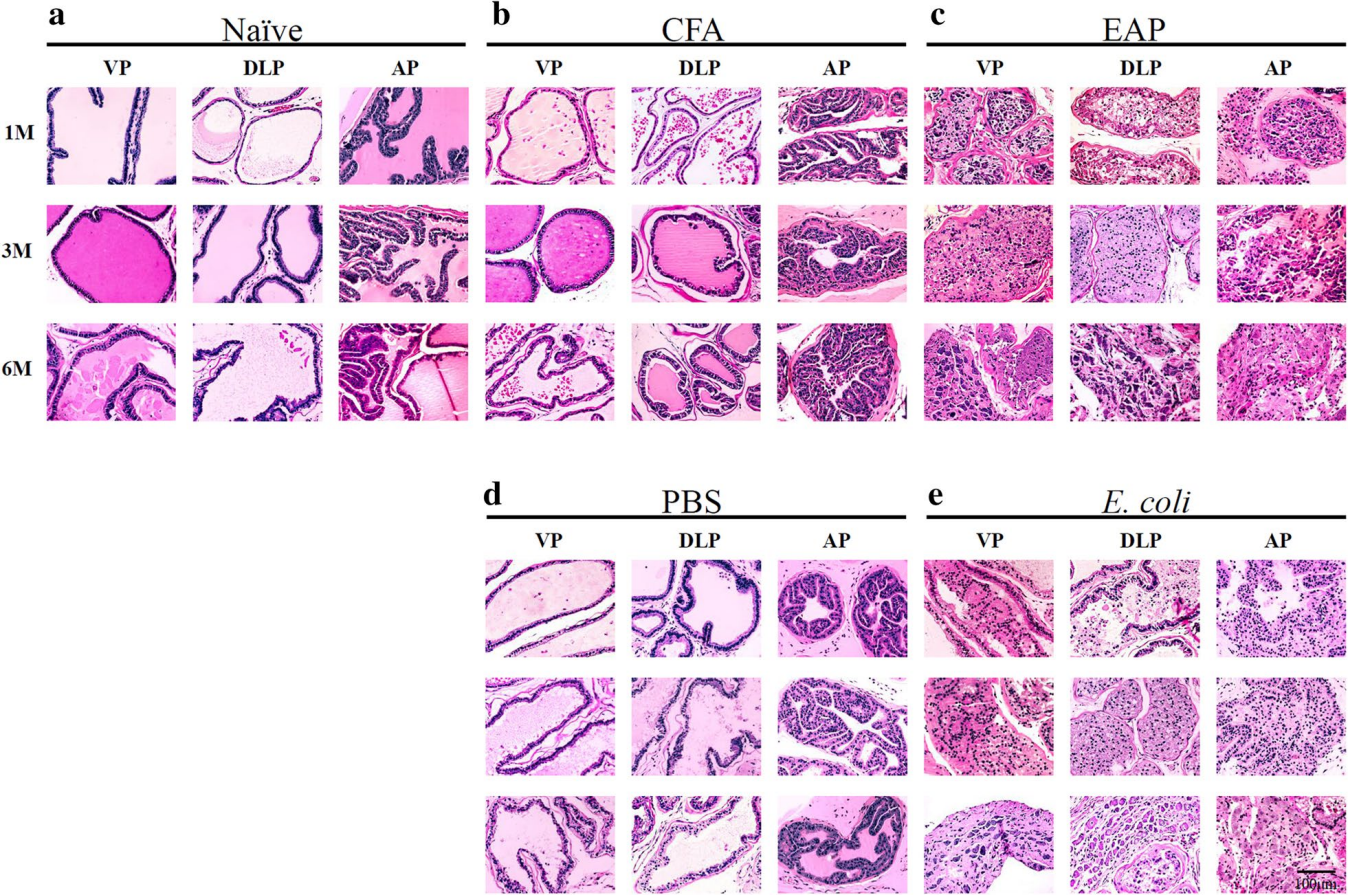
Characteristic images of glandular histopathological findings in ventral prostate (VP), dorsolateral prostate (DLP), and anterior prostate (AP) lobes of mice at 1, 3, and 6 months after immunization or infection (H&E stain, Bar = 100 µm). **a**, **b**, **d** Naïve, CFA, and PBS groups without obvious histopathological changes. CFA group with very mild inflammation at 6 months. **c**, **e** EAP and *E. coli* groups with inflammation characterized by infiltration of lymphocytes, hyperplasia and heteromorphism of glandular epithelium, large and intensely stained nucleolus, and karyokinesis

Prostate inflammation was evaluated histologically (H&E-stained sections) and immunohistochemically. 1 to 6 months after treatment, significantly variable degrees of inflammation were observed among different groups and different prostate lobes (Fig. 1). A month after immunization or bacterial injection, the prostates of the mice in the naïve and control groups (CFA, PBS) showed normal prostatic histology, whereas immune cell infiltrations and significant inflammatory exudate in the glandular cavity were commonly observed in the EAP and *E. coli* groups. Inflammation occurred in the anterior and dorsolateral prostate lobes at high penetrance but less frequently in ventral prostate lobes. Immune cell infiltrations reached the peak on the third month. The CFA group exhibited very mild inflammation on the sixth month possibly due to the urinal reflex-induced chemical and physical trauma.

CD45 is a pan-hematopoietic marker of leukocytes [12, 13]. Immunofluorescence labeling for CD45 revealed the immune cell distribution in the prostate lobes. Significant CD45 immunoreactivity was observed in the prostate lobes in the EAP and *E. coli* groups from 1 to 6 months after treatment (Additional file 1: Figure S1).

**Fig. 2:**
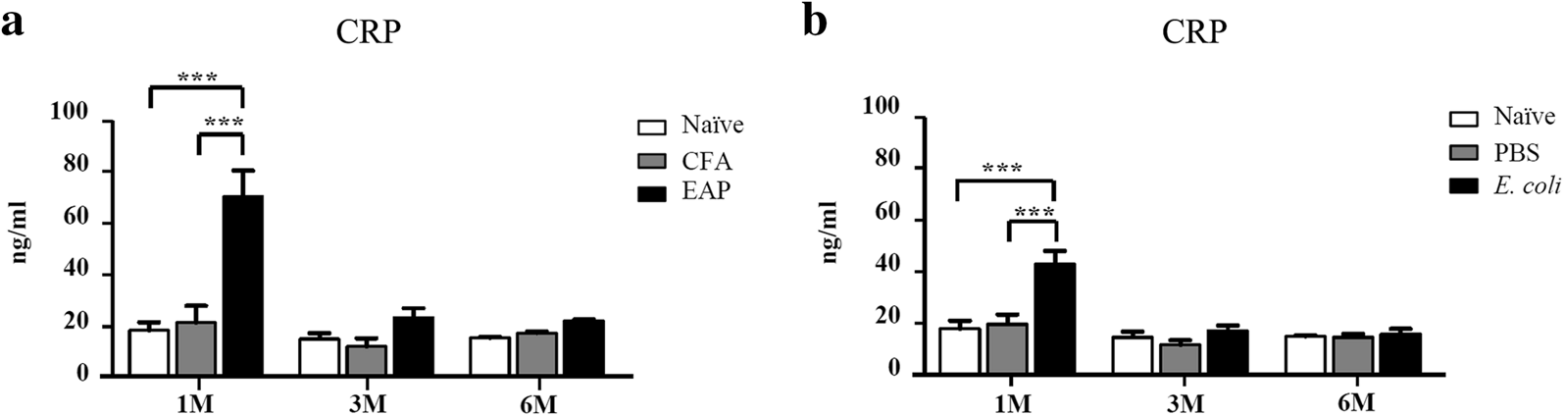
Serum levels of C-reactive protein (CRP) (ng/mL) in mice at 1, 3, and 6 months after immunization or infection. Comparison of serum levels of CRP (ng/mL) among **a** naïve, CFA, and EAP groups and **b** naïve, PBS, and *E. coli* groups (N = 6 per group, data are expressed as mean ± SEM, *P < 0.05, **P < 0.01, ***P < 0.001)

CRP, an acute phase protein, is assessed as a nonspecific marker of inflammation and oxidative stress [14]. The CRP in the EAP and *E. coli* groups was higher than that in the control and naïve groups 1 month after immunization or infection but not after 3 and 6 months (Fig. 2). The CRP serum levels in the EAP and *E. coli* groups increased a month after immunization or infection compared with the levels in the control and naïve groups. However, no statistically significant differences were observed between the EAP and *E. coli* groups for 1 month versus 3 months and 1 month versus 6 months. Likewise, no statistically significant difference was observed between the EAP and *E. coli* groups from one to 6 months. These results indicate a transformation from acute prostatitis to chronic prostatitis in the EAP and *E. coli* groups after immunization or infection.

**Fig. 3:**
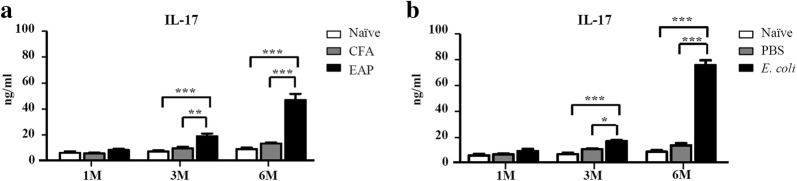
Serum levels of IL-17 (ng/mL) in mice at 1, 3, and 6 months after immunization or infection. **a** Comparison of IL-17 (ng/mL) among naïve, CFA, and EAP groups. **b** Comparison of IL-17 (ng/mL) among naïve, PBS, and *E. coli* groups (N = 6 per group, data are expressed as mean ± SEM, *P < 0.05, **P < 0.01, ***P < 0.001)

Prostatitis was also manifested as an increased serum level of IL-17 (Fig. 3). IL-17 in the EAP group was much higher than that in the CFA group 3 and 6 months after immunization. The same trend was observed for the *E. coli* versus PBS versus naïve group.

### PIA and PIN were commonly observed in chronic prostatitis

Compared with the control and naïve groups, glandular epithelial hyperplasia or atrophy was found in the EAP and *E. coli* groups from 3 to 6 months. PIN shows epithelial hyperplasia characteristics as the enlargement of the glandular lumen, hypertrophic cell, and irregular arrangement (Fig. 1, Additional file 2: Figure S2). PIA is recognized as the presence of atrophic epithelial cells, histological aggressiveness, loss or shrinking of acinar structures, focal atrophy lesions, dilated glandular lumens, and immature epithelial cells next to one another (Fig. 1, Additional file 2: Figure S2). In the high-magnification images, cells enriched with an intermediate phenotype that possessed properties of basal and luminal epithelial cells and debris composed of basal and glandular epithelial cells were found (Additional file 2: Figure S2).

**Fig. 4:**
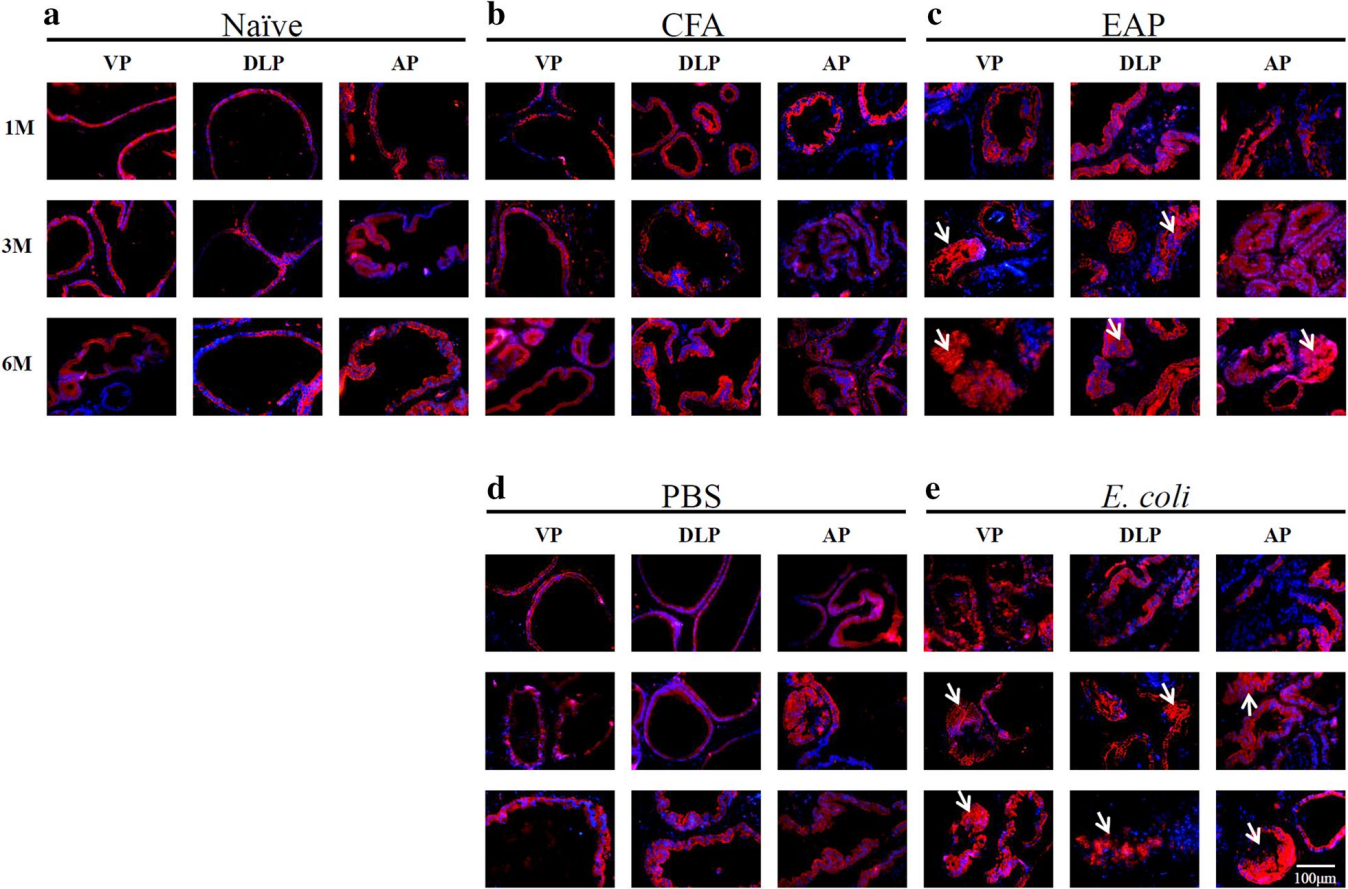
Characteristic monochrome and merged images for CK8 (red) in ventral prostate (VP), dorsolateral prostate (DLP), and anterior prostate (AP) lobes of mice at 1, 3, and 6 months after immunization or infection. Nuclei are counterstained with DAPI (blue). **a**, **b**, **d** Naïve, CFA, and PBS groups. A single layer of luminal cells is present, and no proliferating cells are observed. **c**, **e** EAP and *E. coli* groups. Multiple layers of luminal cells positive for CK8+ (in red) are observed at the third and sixth month of modeling

**Fig. 5:**
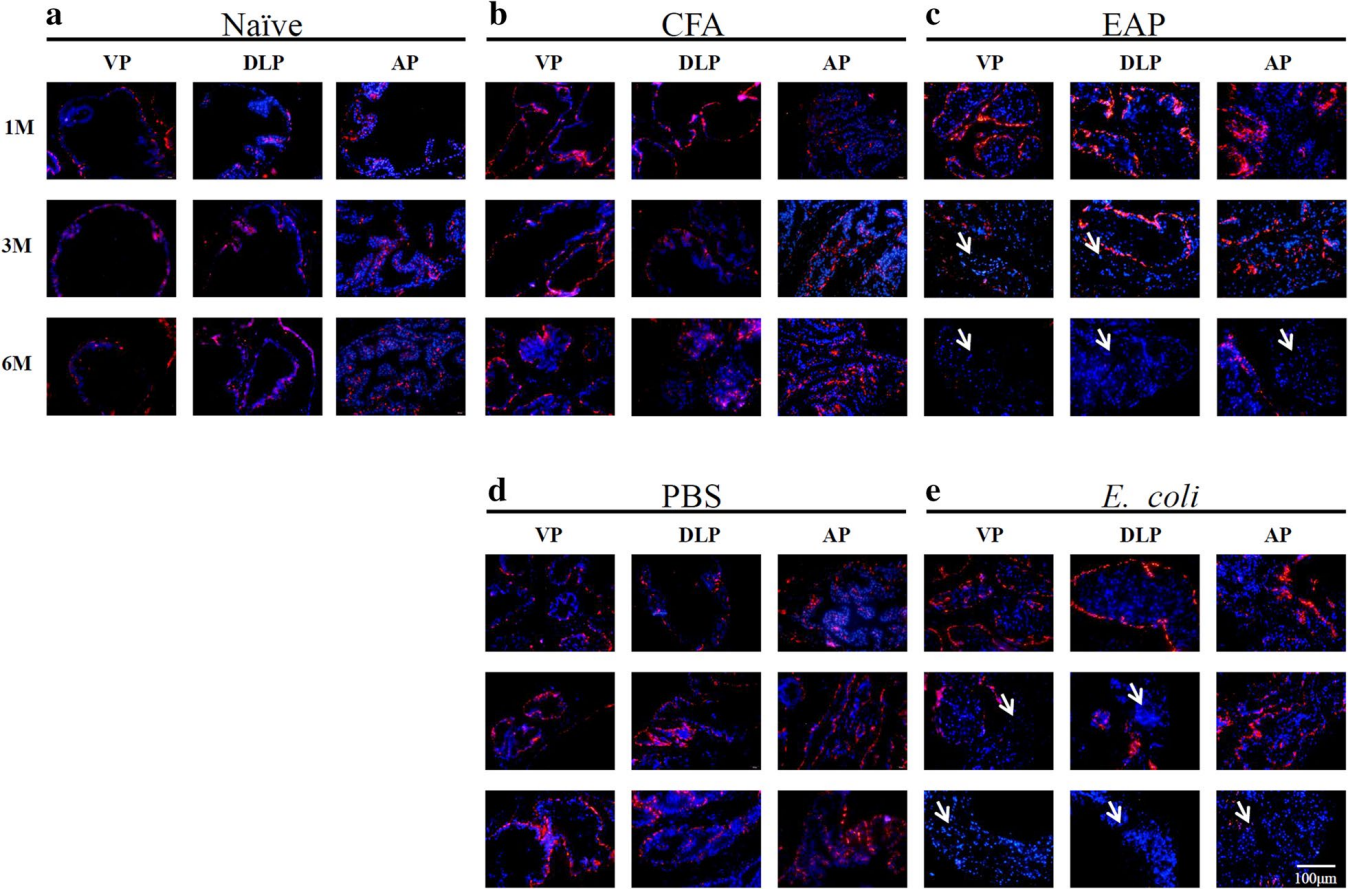
Characteristic monochrome and merged images for CK5 (red) in ventral prostate (VP), dorsolateral prostate (DLP), and anterior prostate (AP) lobes of mice at 1, 3, and 6 months after immunization or infection. Nuclei are counterstained with DAPI (blue). **a**, **b**, **d** Naïve, CFA, and PBS groups. CK5+ (red) can be observed from 1 to 6 months. **c** EAP and *E. coli* groups. Basal cells positive for CK5+ (red) are observed in the first month, obviously decrease in the third month, and almost disappear in the sixth month

CK5/CK8 immunofluorescence and p63 immunohistochemistry were applied to further distinguish the cell type (Figs. 4, 5). Basal cells with good cell plasticity display CK5 and p63, and functional luminal cells show the expression of CK8 [15]. Single layers of luminal cells (CK8+) and basal cells (CK5+/p63+) were observed in the naïve and control groups. By contrast, a dramatic decrease in basal cells (CK5+/p63+) and accumulation of luminal epithelial cells (CK8+) were found in the inflamed prostatic lobes for EAP and *E. coli* groups. On the sixth month, nearly all of the CK5 stain disappeared in the EAP and *E. coli* groups (Fig. 5), which meant that the basal cells were damaged and transformed into different luminal cells.

Interstitial tissue hyperplasia was found, and Masson’s trichrome staining showed an increase in collagen deposition (blue staining) and a reduction in smooth muscle cells (red staining) in the prostate stromal of the prostate lobes of EAP and *E. coli* groups from 3 to 6 months. Fibrosis increased with time (Additional file 3: Figure S3A). After quantification of the collagen fibers by Image Pro Plus 6.0 IOD, the EAP group showed a remarkable increase in the PBS group 3 and 6 months after treatment when compared with the CFA group (Additional file 3: Figure S3B). The same trend was observed for *E. coli* versus PBS or versus naïve group (Additional file 3: Figure S3C). Significant differences in muscle fibers were also noted among the EAP, CFA, and naïve groups from 1 to 6 months (Additional file 3: Figure S3D). Similar differences were found among the *E. coli*, PBS, and naïve groups (Additional file 3: Figure S3E).

### Chronic prostatitis induced cell apoptosis

We observed changes in the organelle ultrastructure through transmission electron microscopy. In the EAP and *E. coli* groups, changes in cellular organelles included swollen mitochondria with an irregular membrane, loss of mitochondrial membranes, disrupted mitochondrial granules, and irregular nuclear membranes. The CFA, PBS, and naïve groups showed a normal organelle ultrastructure, which indicates a tendency toward apoptosis in the EAP and *E. coli* groups (Additional file 4: Figure S4).

Heat shock protein 60 (HSP60) is a novel apoptotic and prostate cancer prognostic marker that is involved in the regulation of HIF-1α protein stability and associated with prostate cancer progression [16, 17]. After prostate extract protein immunization or bacterial infection, the expression of HSP60 in the prostate lobes increased significantly from 1 to 6 months (Additional file 5: Figure S5A). Comparison of the IOD values of EAP, CFA, and naïve groups revealed significant differences (Additional file 5, Figure S5B). The same trend was apparent among *E. coli*, PBS, and naïve groups (Additional file 5: Figure S5C).

### Inflammatory prostate microenvironment caused chronic pelvic pain

Referred hyperalgesia and tactile allodynia were examined by applying von Frey filaments to the pelvic region. The EAP and *E. coli* groups developed chronic pelvic pain, as indicated by decreased pain threshold responses to a stimulus in the pelvic region. Higher pain thresholds were observed in the EAP group compared with the CFA group. The same pattern was noted for the EAP versus naïve group (Additional file 6: Figure S6A). A similar trend was found for the *E. coli* group compared with the PBS group for 6 months (all *P *< 0.05) (Additional file 6: Figure S6B).

To investigate how the inflammatory prostatic microenvironment influenced the histology of other organs in the reproductive system, H&E staining was performed in the testis, epididymis, and foreskin gland of the mice in the EAP and *E. coli* groups 6 months after immunization or infection. The results showed variable degrees of inflammation in the testis, epididymis, and foreskin gland compared with the control and naïve groups (Additional file 7: Figure S7).

### Chronic inflammation resulted in somatic mutations

The exon capture baits targeted 99.5% of genes in the GATK database mm10. Gene exon variants were filtered for quality according to several criteria (Additional file 8: Table S1). Variations were observed broadly across the genome in the naïve, EAP, CFA, *E. coli* and PBS groups at different time points. The mutation rate between different groups did not obviously differ. The gene exon variants ranged from 121 to 172 in different groups at different points, which included frameshift mutation, non-frameshift mutation, synonymous mutation, non-synonymous mutation, stop gain, and stop loss. The affected gene had no obvious difference in different groups (Additional file 9: Table S2, Additional file 10: Figure S8). We focused our analysis on genes with high or moderate impact variants, such as non-synonymous, frameshift mutation, stop gain, and stop loss, that are more likely to result in significant functional changes. As a result, several mutation genes were distinguished in the EAP or *E. coli* groups. Mutations affected the EAP group but were absent in the naïve or CFA group at 1 month (including Nacad, Prg4, and Zfp148), at 3 months (including Cyp3a41b, Glrp1, Gm572, Olfr849, and Sult2a6), and at six months (including Hic1 and Mfge8) (Table 1, Additional file 11: Table S3). Mutations affected the *E. coli* group but were absent in the naïve or PBS group at 1 month (including Gm21708, Gm572, H2-Q6, Phactr4, Rsf1, Slc5a4b, and Zfp148), at 3 months (including Csprs, Gm572, and Slc5a4b), and at 6 months (including Cdh11, Mfge8, milk, and fat) (Table 2, Additional file 12: Table S4). Beyond the gene difference, genomic variants in EAP and *E. coli* groups proved to be related. The most frequently mutated genes were Gm572, Slc5a4b, Zfp148, and Mfge8 (Additional file 13: Figure S9), which is genes that are recurrently mutated in chronic prostate inflammation.

**Table 1:** Gene exon variants in EPA group but absent in CFA or naïve group

	Chromosome	Gene	EAP (count)	CFA (count)	Naïve (count)
1 month	11	Nacad	3	0	0
	1	Prg4	8	0	0
	16	Zfp148	3	0	0
3 month	5	Cyp3a41b	3	0	0
	1	Glrp1	3	0	0
	4	Gm572	3	0	0
	9	Olfr849	3	0	0
	7	Sult2a6	4	0	0
6 month	11	Hic1	3	0	0
	7	Mfge8	3	0	0

**Table 2:** Gene exon variants in EPA group but absent in CFA or naïve group

	Chromosome	Gene	*E. coli* (count)	PBS (count)	Naïve (count)
1 month	Y	Gm21708	3	0	0
	4	Gm572	3	0	0
	17	H2-Q6	6	0	0
	4	Phactr4	3	0	0
	7	Rsf1	3	0	0
	10	Slc5a4b	3	0	0
	16	Zfp148	3	0	0
3 month	1	Csprs	4	0	0
	4	Gm572	3	0	0
	10	Slc5a4b	3	0	0
6 month	8	Cdh11	6	0	0
	7	Mfge8	3	0	0

## Discussion

We established immune and bacterial prostatitis mouse models induced by prostate extract protein immunization injection or *E. coli* infection. CRP in the EAP and *E. coli* groups was higher than that in the control and naïve groups 1 month after immunization or infection. Serum IL-17 levels were elevated at 3 months and continued until 6 months. High inflammation cytokine helps strengthen the immune response in the initiation of inflammation, but long-term high concentrations of inflammation cytokines are harmful. Previous studies have shown that inflammatory cytokines, including CRP [14, 18], IL-6 [18–20], and IL-17 [21, 22], are positively associated with the risk of prostate cancer. Research has proven that a high level of inflammation cytokine results in basal-to-luminal differentiation in mouse models [23]. Human and rodent basal cells possess the capacity for multiline age differentiation, and transfer of different basal cells is possible as preneoplastic lesions of prostate cancer [24–26].

Continuous inflammation leads to base membrane damage, epithelium atrophy, and abnormal proliferation [9, 15, 27]. In our present study, PIA and PIN were commonly observed in the EAP and *E. coli* groups after 3 months. Furthermore, a dramatic decrease in basal cells (CK5+/p63+) and accumulation of luminal epithelial cells (CK8+) were found in the inflamed prostatic lobes for both EAP and *E. coli* groups, especially at the sixth-month check point. This result means that the base cells were damaged or transformed into luminal cells. The PIA areas were enriched with cells phenotypically intermediate between basal and secretory cells. These cells expressed luminal epithelial cell marker CK8 and CK18, but they also expressed basal cell marker CK5 [28, 29]. These cells exhibited high epithelial proliferation rates and infiltration by inflammatory cells [30]. PIA or PIN accumulated genetically changed lesions, such as GSTP1 hypermethylation [31–33] or MYC overexpression [34]. PIA lesions may give rise to carcinoma either directly or by developing into high-grade PIN [28, 31, 35]. In general, we think that chronic inflammation induced PIA and PIN cancer precursor lesions in this study, which are closely associated with prostate cancer.

Chronic pelvic pain is the most common symptom in chronic prostatitis patients. The basis of pelvic pain could be local neuroinflammation induced by local cytokine production from autoimmune/inflammatory processes [36, 37]. Compared with the control and naïve groups, the EAP and *E. coli* groups showed significant increases in the pain threshold from 1 to 6 months. The phenomenon of prostatitis resulted in termed cross-organ sensitization. These findings are consistent with previous reports that inflammation in the prostate or bladder can induce the chronic prostatitis/chronic prostatic pain (CP/CPPP) syndrome [27]. The cross-organ sensitization symptom has also been observed in rat bladder inflammation models [38].

Prostate cancer is a tumor that is closely related to gene mutation. Common genetic alterations in prostate cancer include HPCI mutation [39], losses or down-regulation of the tumor suppressor protein NKX3.1 [40, 41], loss or mutation of PTEN [42, 43], TMPRSS2-AR or ERG1-AR gene fusion [44–46], and AR upregulation [47]. Through GWE sequencing, we provided evidence that chronic inflammation induces somatic exon mutation in the prostate. Several mutation genes were distinguished in the EAP and CFA groups from the naïve, CFA, or PBS control groups. This is the first study to provide evidence that prostatitis induces somatic cell mutation in mouse models.

Mutations affected Hic1 in the EAP group but were absent in the naïve or CFA group at 6 months. This gene functions as a growth regulator and tumor repressor. Hypermethylation of HIC1 promoter results in the loss of its repressive function, and it is also a core gene mutation in prostate cancer [48]. Moreover, impairment of the HIC1 expression in the PCa cell could accelerates EMT (epithelial–mesenchymal transition) and promotes migration and metastasis. HIC1 rescue would significantly inhibit proliferation, migration, and invasion and induce apoptosis [49, 50].

Beyond the gene difference, genomic variants in the EAP and *E. coli* groups proved to be related. The most frequently mutated genes were Gm572, Slc5a4b, Zfp148, and Mfge8. Mfge8 is a bridge protein for membrane-encased collection of proteins and triglycerides; it can facilitate efferocytosis and promote an innate immunity reaction by promoting macrophage polarization [51]. The serum level of Mfge8 is higher in prostate cancer patients than in control subjects [52].

Zfp148 is another gene mutation that came into notice. Zfp148 is a member of the Kruppel family of zinc finger DNA-binding proteins, and it interacts physically with the tumor suppressor protein 53 (P53). Zfp148 deficiency causes lung maturation defects and lethality in newborn mice, and this defect can be addressed by deleting Zfp148 binding P53 or providing antioxidant treatment [53]. Zfp148-deficient fibroblasts exhibit proliferative arrest [53]. Appropriate Zfp148 expression is important to cellular proliferation, embryogenesis, differentiation, growth arrest, and apoptosis [54].

To our knowledge, this study is the first to explore the roles of inflammation in prostate somatic gene mutation. However, this study has inevitable limitations. First, no prostate cancer outcome was observed, and only PIA and PIN were characterized. Second, exon sequences were used to detect somatic mutation in the prostate. Common mutations in prostate cancer, such as promoter hypermethylation, multi-copy gene segment, and gene fusion, might have been easily missed. Third, due to the lack of double staining CK8 and CK5, we could not distinguish intermediate cells that had properties of both basal and luminal epithelial cells in the PIA area.

## Conclusion

Chronic prostatitis induced by prostate extract protein immunization or *E. coli* infection causes a reactive prostatic inflammation microenvironment and results in tissue damage, aberrant atrophy, hyperplasia, and somatic genome mutation. It also accelerates the initiation and progression of preneoplastic prostate lesions in C57BL/6 mice.

## Additional files

**Additional file 1: Figure S1.** Characteristic images of immune cell distribution marked by CD45 immunolabeling (red fluorescence) in ventral prostate (VP), dorsolateral prostate (DLP), and anterior prostate (AP) lobes of mice at 1, 3, and 6 months after immunization or infection. **A**, **B**, **D:** Naïve, CFA, and PBS groups without immune cells at 1 month. CFA and PBS groups have few immune cells at 6 months. **C**, **E:** EAP and *E. coli* groups have immune cells in stroma in the beginning of modeling and even in the prostate glands 6 months after treatment.

**Additional file 2: Figure S2.** Characteristic monochrome and merged images for p63 in ventral prostate (VP), dorsolateral prostate (DLP), and anterior prostate (AP) lobes of mice at 1, 3, and 6 months after immunization or infection. **A**, **B**, **D:** The naïve group has a single layer of basal cells, and no proliferating cells are observed. **C**, **E:** EAP and *E. coli* groups have multiple layers of basal cells positive for p63 in the beginning of modeling; the absence of basal cells in local prostate lobes appeared from 3 to 6 months after treatment.

**Additional file 3: Figure S3.** Masson’s trichrome stain pictures of pathological fibrosis in ventral prostate (VP), dorsolateral prostate (DLP), and anterior prostate (AP) lobes of mice at 1, 3, and 6 months after immunization or infection. The red staining shows smooth muscle cells; the blue staining shows collagenous stroma. **A:** Naïve group without pathological fibrotic changes, CFA group without pathological fibrotic changes, EAP group with collagen deposition and dense fibrosis, PBS group without pathological fibrotic changes, and *E. coli* group with collagen deposition and dense fibrosis. Comparison of collagen fibers (average IOD in %) among **B:** naïve, CFA, and EAP groups; **C:** naïve, PBS, and *E. coli* groups. Comparison of muscle fibers (average IOD in %) among **D:** naïve, CFA, and EAP groups; **E:** naïve, PBS, and *E. coli* groups (N = 6 per group, data are expressed as mean ± SEM, *P < 0.05, **P < 0.01, ***P < 0.001).

**Additional file 4: Figure S4.** Transmission electron microscope images at 1, 3, and 6 months after immunization or infection. Naïve, CFA, and PBS groups without changes in the organelle ultrastructure. EAP and *E. coli* group with changes in the organelle ultrastructure, including disrupted mitochondrial granules, enlarged endoplasmic reticulum, degradation of mitochondrial cristae, accumulation of cytoplasmic lysosomes, and irregular nuclear membrane.

**Additional file 5: Figure S5.** Immunohistochemical Hsp60 expression in anterior prostate (AP), dorsolateral prostate (DLP), and ventral prostate (VP) lobes of mice at 1, 3, and 6 months after immunization or infection. **A:** Naïve, CFA, and PBS groups with no clear positive signals. EAP and *E. coli* groups with widespread strong positive signals in the cytoplasm. Comparison of Hsp60 (average IOD in %) among **B:** naïve, CFA, and EAP groups and **C:** naïve, PBS, and *E. coli* groups (N = 6 per group, data are expressed as mean ± SEM, *P < 0.05, **P < 0.01, ***P < 0.001).

**Additional file 6: Figure S6.** Comparison of pelvic pain assessment among different groups. Proportion analysis (%, left y axis) and average levels (right y axis) of pain thresholds in **A:** naïve, CFA, and EAP groups and **B:** naïve, PBS, and *E. coli* groups (N = 6 per group, data are expressed as mean ± SEM, *P < 0.05, **P < 0.01, ***P < 0.001).

**Additional file 7: Figure S7.** Characteristic images of histopathological findings in the testis, epididymis, and foreskin gland of mice 6 months after immunization or infection (H&E staining, Bar = 100 µm). Naïve, CFA, and PBS groups without histopathological changes. EAP and *E. coli* groups with inflammation characterized by infiltration of lymphocytes and hyperplasia.

**Additional file 8: Table S1.** SNP and index exclusion criteria.

**Additional file 9: Table S2.** Gene exon variants in CFA, EPA, *E. coli*, PBS and naive group at 1, 3, 6 month after injection or infection.

**Additional file 10: Figure S8.** Heat maps showing strain-specific profiles of the number of exome variants per 10-Mb bin across every chromosome in naïve, CFA, EAP, PBS, and *E. coli* groups at 1, 3, and 6 months after immunization or infection. The darker the shade of blue is, the stronger the correlation is.

**Additional file 11:Table S3.** Mutations affected the EAP group but were absent in the naïve or CFA group at one, three and six months.

**Additional file 12: Table S4.** Mutations affected the E.coli group but were absent in the naïve or PBS group at one, three and six months.

**Additional file 13: Figure S9.** Venn diagram showing the overlap mutation gene among naïve, CFA, EPA, PBS, and *E. coli* groups at different time points of 1, 3, and 6 months.

## Data Availability

All data generated or analyzed during this study are included in this published article (and its additional information files).

## Abbreviations

EAP: prostate extract protein
CFA: complete Freund’s adjuvant
PBS: phosphate buffer saline
CRP: C-reactive protein
HSP60: heat shock protein 60
PIA: proliferative inflammatory atrophy
PIN: prostatic intraepithelial neoplasia
GWE: genome-wide exome
SEM: standard error of the mean
