# Supplementary figures and images for: Chronic prostatitis alters the prostatic microenvironment and accelerates preneoplastic lesions in C57BL/6 mice
Source: Biol Res. 2019 May 14;52:30. doi: 10.1186/s40659-019-0237-4 (PMC6518623; doi:10.1186/s40659-019-0237-4)

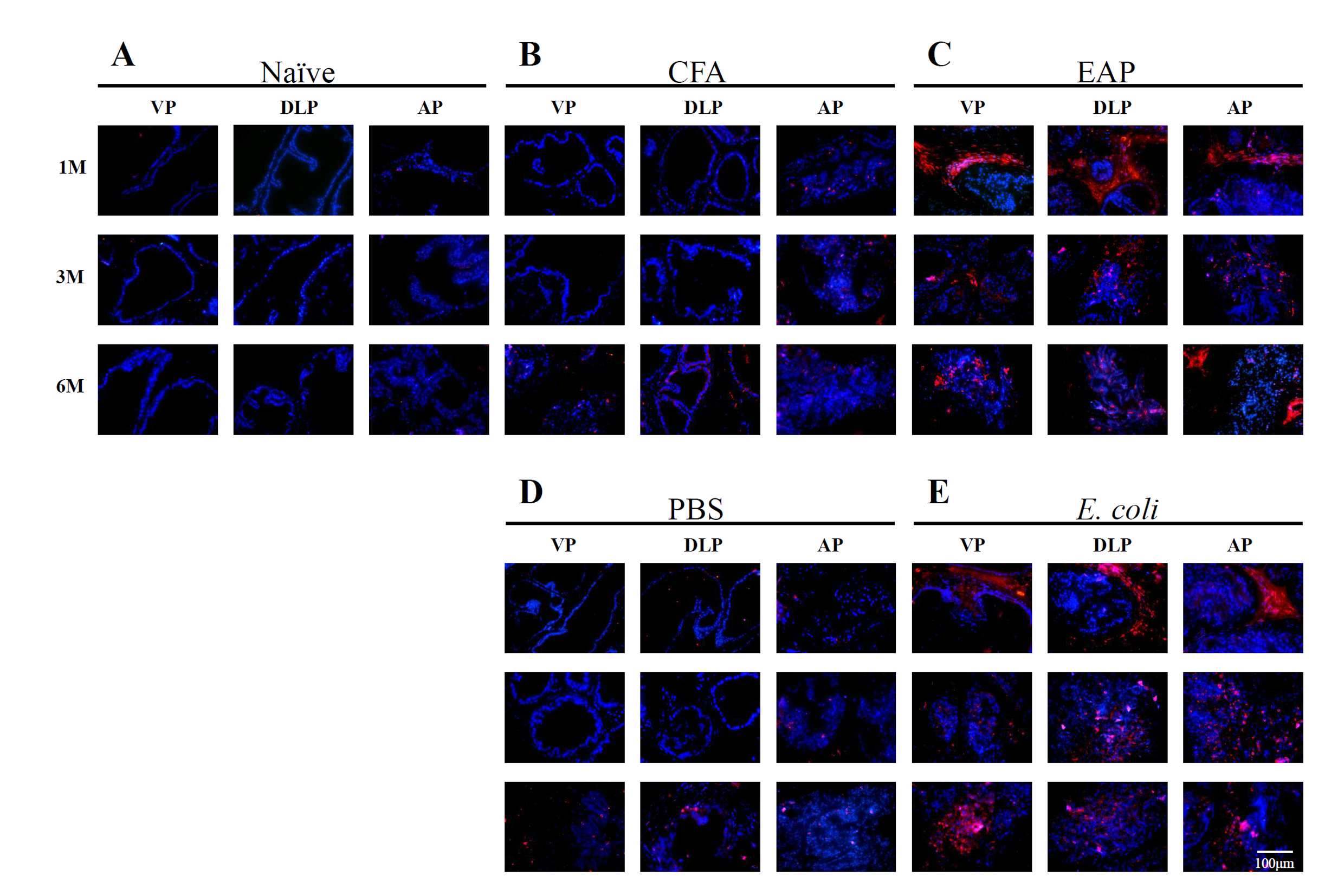

Supplement: Supplementary file 1 — Additional file 1: Figure S1. Characteristic images of immune cell distribution marked by CD45 immunolabeling (red fluorescence) in ventral prostate (VP), dorsolateral prostate (DLP), and anterior prostate (AP) lobes of mice at 1, 3, and 6 months after immunization or infection. A, B, D: Naïve, CFA, and PBS groups without immune cells at 1 month. CFA and PBS groups have few immune cells at 6 months. C, E: EAP and E. coli groups have immune cells in stroma in the beginning of modeling and even in the prostate glands 6 months after treatment. [file 40659_2019_237_MOESM1_ESM.tif]

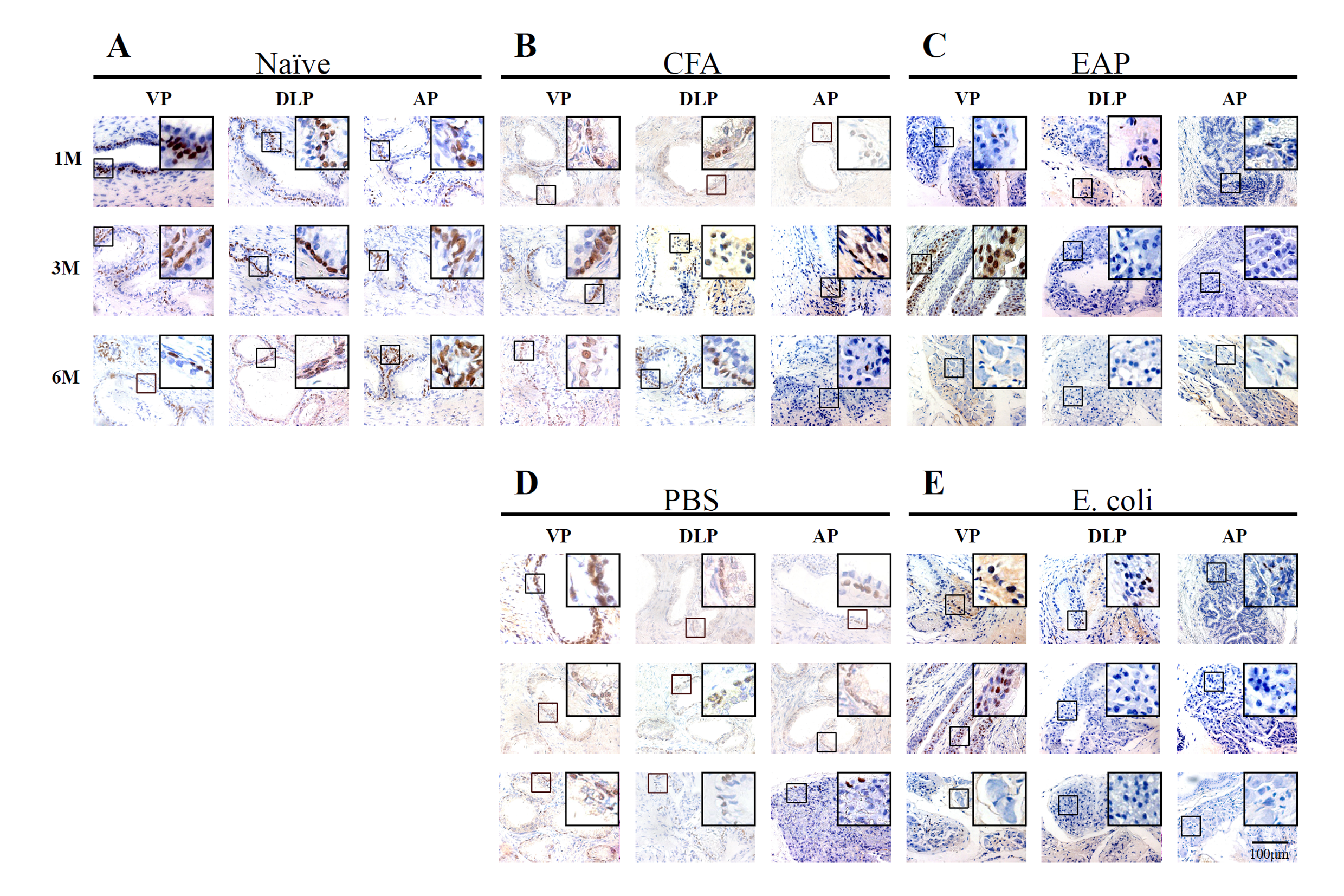

Supplement: Supplementary file 2 — Additional file 2: Figure S2. Characteristic monochrome and merged images for p63 in ventral prostate (VP), dorsolateral prostate (DLP), and anterior prostate (AP) lobes of mice at 1, 3, and 6 months after immunization or infection. A, B, D: The naïve group has a single layer of basal cells, and no proliferating cells are observed. C, E: EAP and E. coli groups have multiple layers of basal cells positive for p63 in the beginning of modeling; the absence of basal cells in local prostate lobes appeared from 3 to 6 months after treatment. [file 40659_2019_237_MOESM2_ESM.tif]

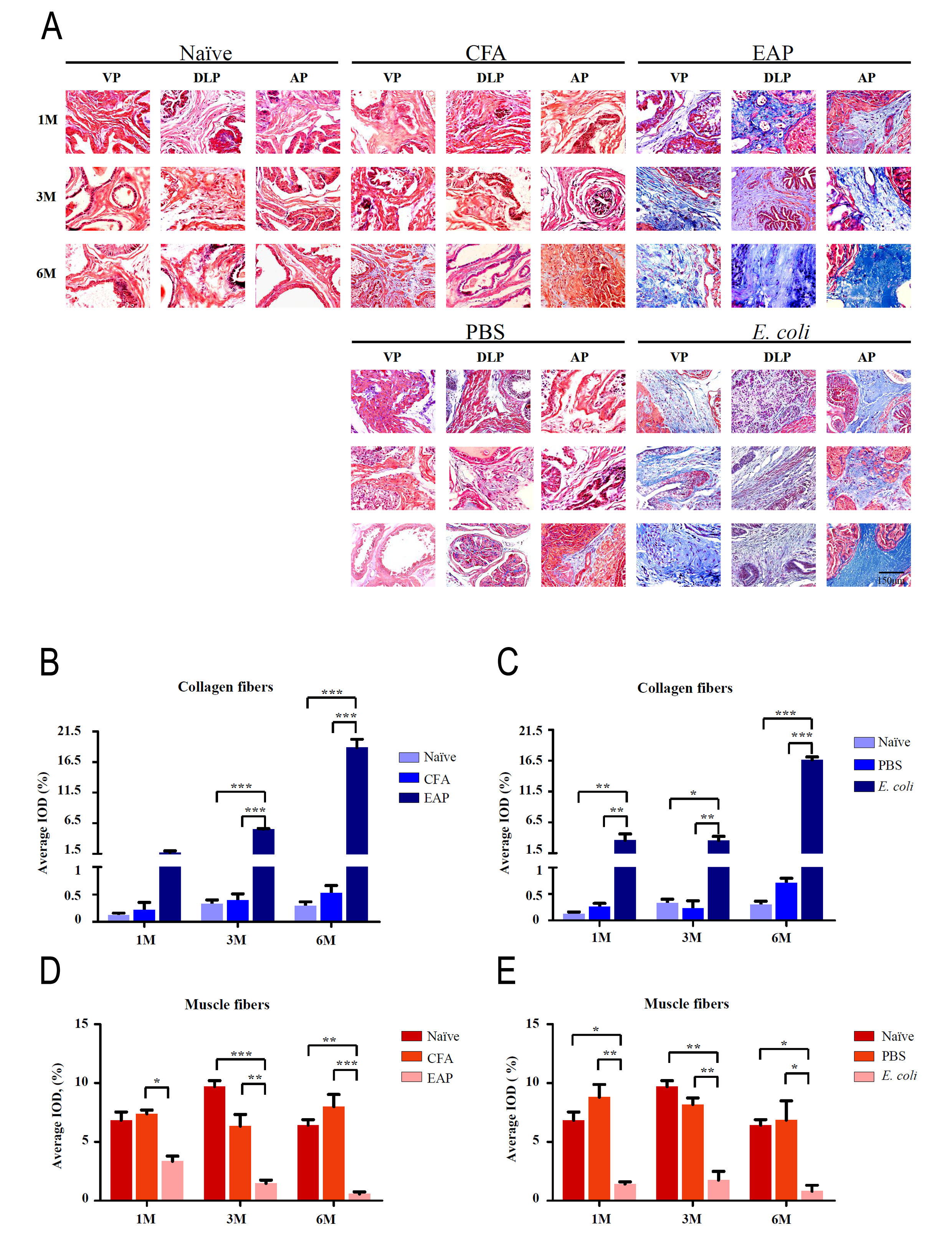

Supplement: Supplementary file 3 — Additional file 3: Figure S3. Masson’s trichrome stain pictures of pathological fibrosis in ventral prostate (VP), dorsolateral prostate (DLP), and anterior prostate (AP) lobes of mice at 1, 3, and 6 months after immunization or infection. The red staining shows smooth muscle cells; the blue staining shows collagenous stroma. A: Naïve group without pathological fibrotic changes, CFA group without pathological fibrotic changes, EAP group with collagen deposition and dense fibrosis, PBS group without pathological fibrotic changes, and E. coli group with collagen deposition and dense fibrosis. Comparison of collagen fibers (average IOD in %) among B: naïve, CFA, and EAP groups; C: naïve, PBS, and E. coli groups. Comparison of muscle fibers (average IOD in %) among D: naïve, CFA, and EAP groups; E: naïve, PBS, and E. coli groups (N = 6 per group, data are expressed as mean ± SEM, *P < 0.05, **P < 0.01, ***P < 0.001). [file 40659_2019_237_MOESM3_ESM.tif]

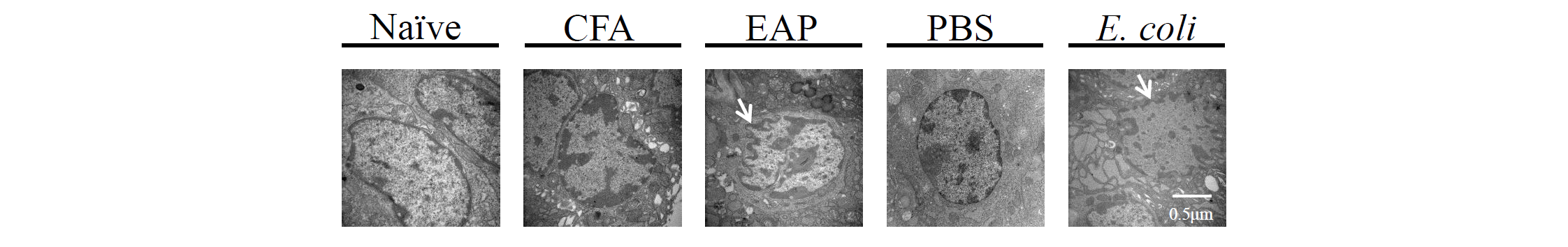

Supplement: Supplementary file 4 — Additional file 4: Figure S4. Transmission electron microscope images at 1, 3, and 6 months after immunization or infection. Naïve, CFA, and PBS groups without changes in the organelle ultrastructure. EAP and E. coli group with changes in the organelle ultrastructure, including disrupted mitochondrial granules, enlarged endoplasmic reticulum, degradation of mitochondrial cristae, accumulation of cytoplasmic lysosomes, and irregular nuclear membrane. [file 40659_2019_237_MOESM4_ESM.tif]

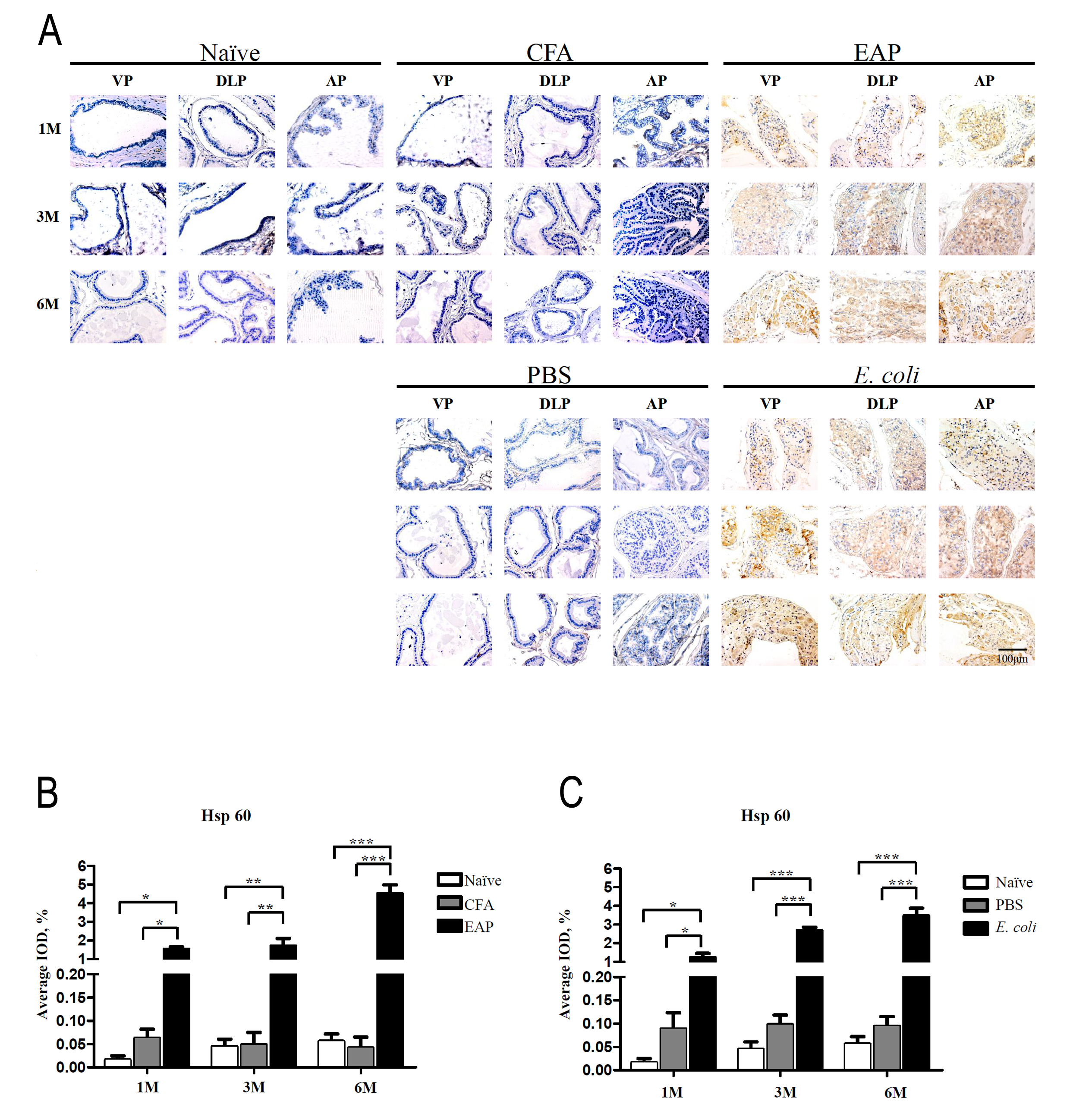

Supplement: Supplementary file 5 — Additional file 5: Figure S5. Immunohistochemical Hsp60 expression in anterior prostate (AP), dorsolateral prostate (DLP), and ventral prostate (VP) lobes of mice at 1, 3, and 6 months after immunization or infection. A: Naïve, CFA, and PBS groups with no clear positive signals. EAP and E. coli groups with widespread strong positive signals in the cytoplasm. Comparison of Hsp60 (average IOD in %) among B: naïve, CFA, and EAP groups and C: naïve, PBS, and E. coli groups (N = 6 per group, data are expressed as mean ± SEM, *P < 0.05, **P < 0.01, ***P < 0.001). [file 40659_2019_237_MOESM5_ESM.tif]

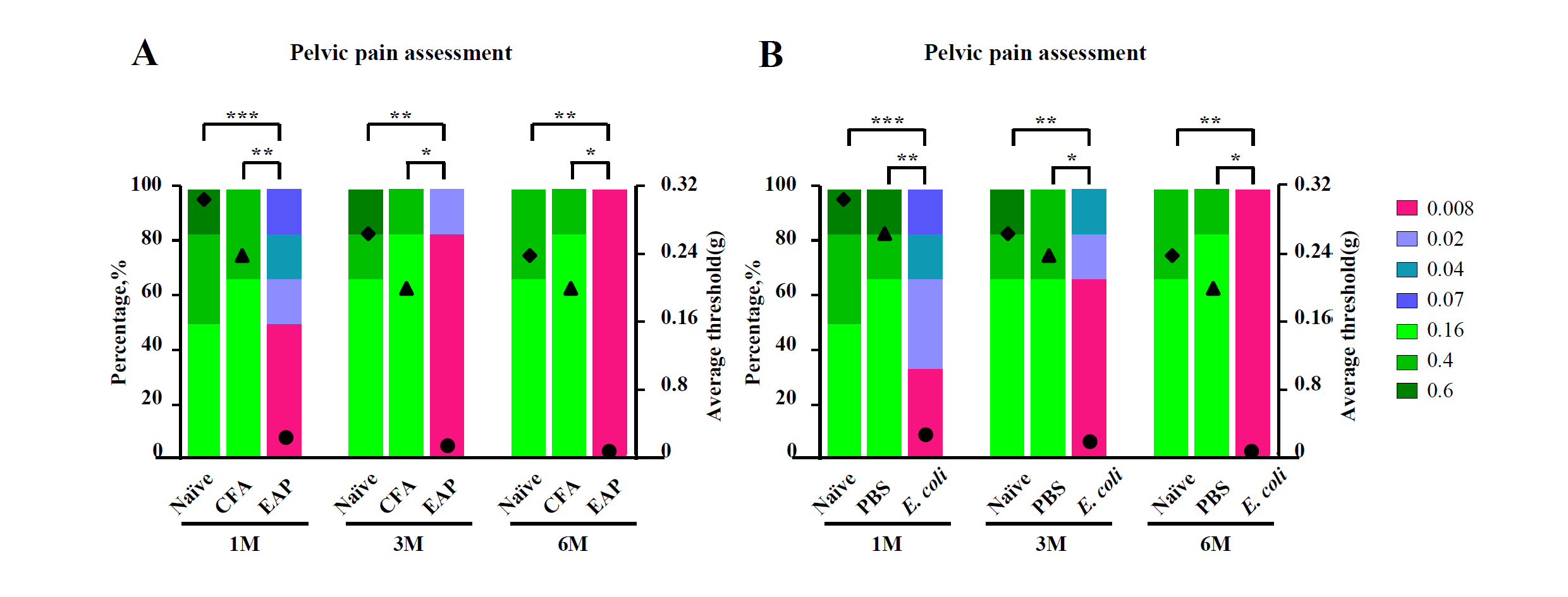

Supplement: Supplementary file 6 — Additional file 6: Figure S6. Comparison of pelvic pain assessment among different groups. Proportion analysis (%, left y axis) and average levels (right y axis) of pain thresholds in A: naïve, CFA, and EAP groups and B: naïve, PBS, and E. coli groups (N = 6 per group, data are expressed as mean ± SEM, *P < 0.05, **P < 0.01, ***P < 0.001). [file 40659_2019_237_MOESM6_ESM.tif]

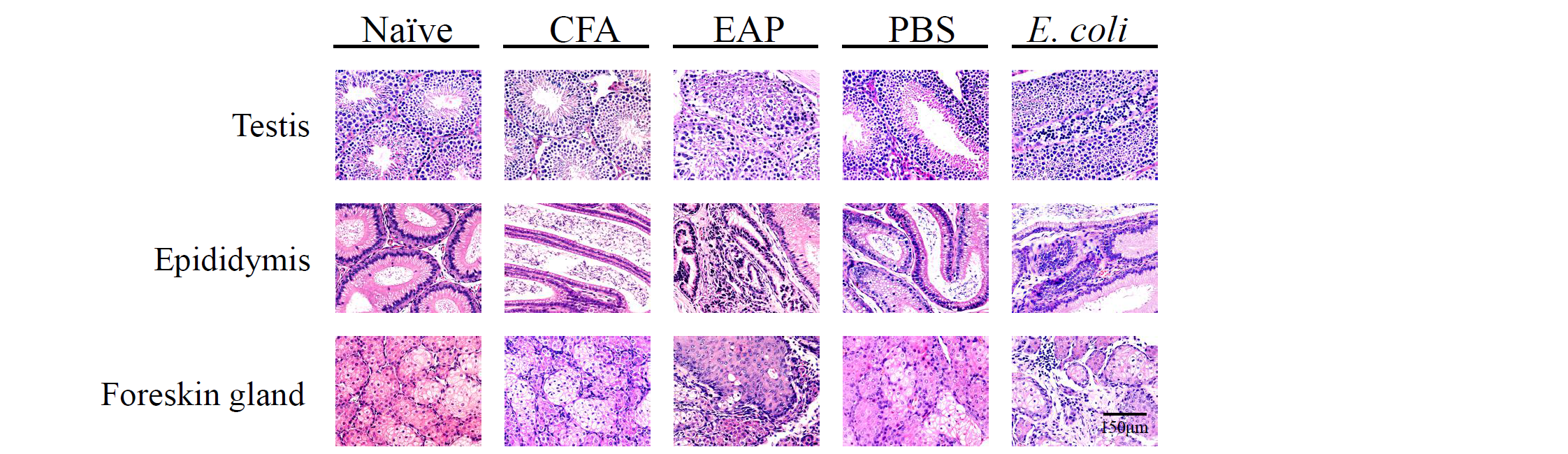

Supplement: Supplementary file 7 — Additional file 7: Figure S7. Characteristic images of histopathological findings in the testis, epididymis, and foreskin gland of mice 6 months after immunization or infection (H&E staining, Bar = 100 µm). Naïve, CFA, and PBS groups without histopathological changes. EAP and E. coli groups with inflammation characterized by infiltration of lymphocytes and hyperplasia. [file 40659_2019_237_MOESM7_ESM.tif]

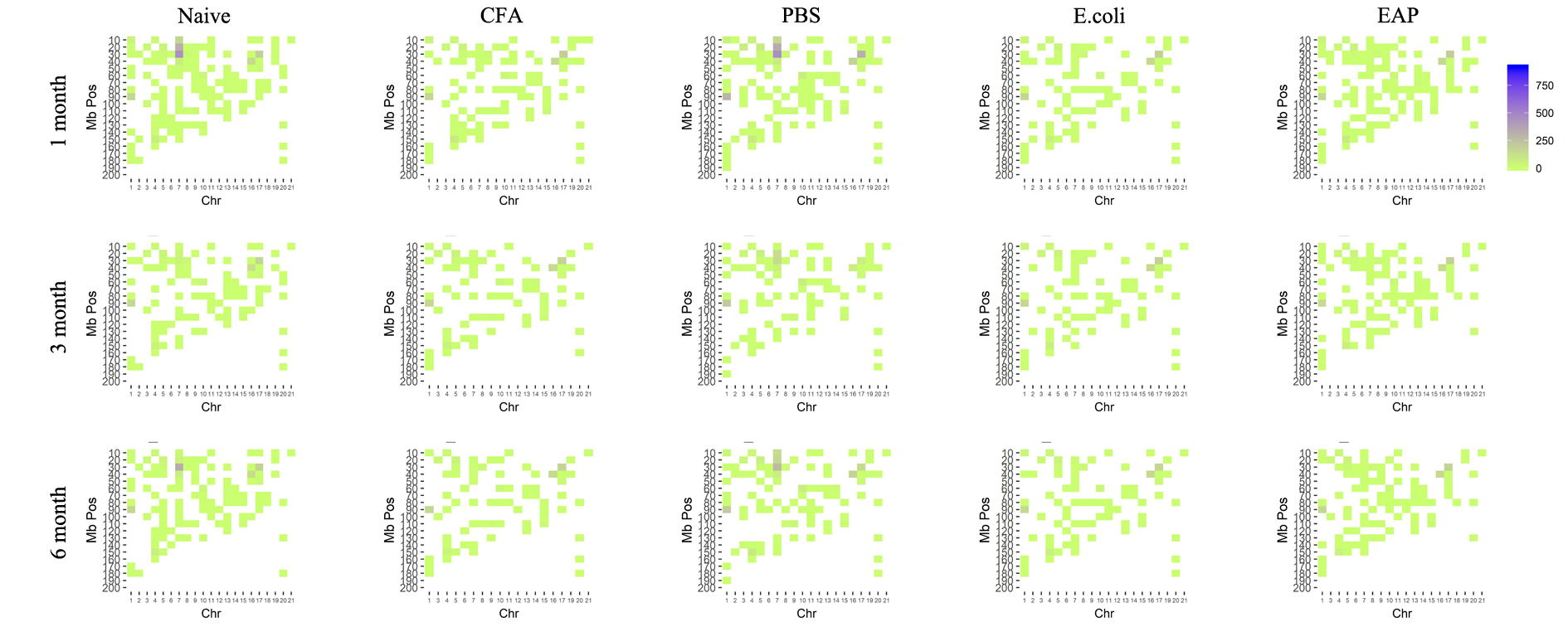

Supplement: Supplementary file 10 — Additional file 10: Figure S8. Heat maps showing strain-specific profiles of the number of exome variants per 10-Mb bin across every chromosome in naïve, CFA, EAP, PBS, and E. coli groups at 1, 3, and 6 months after immunization or infection. The darker the shade of blue is, the stronger the correlation is. [file 40659_2019_237_MOESM10_ESM.tif]

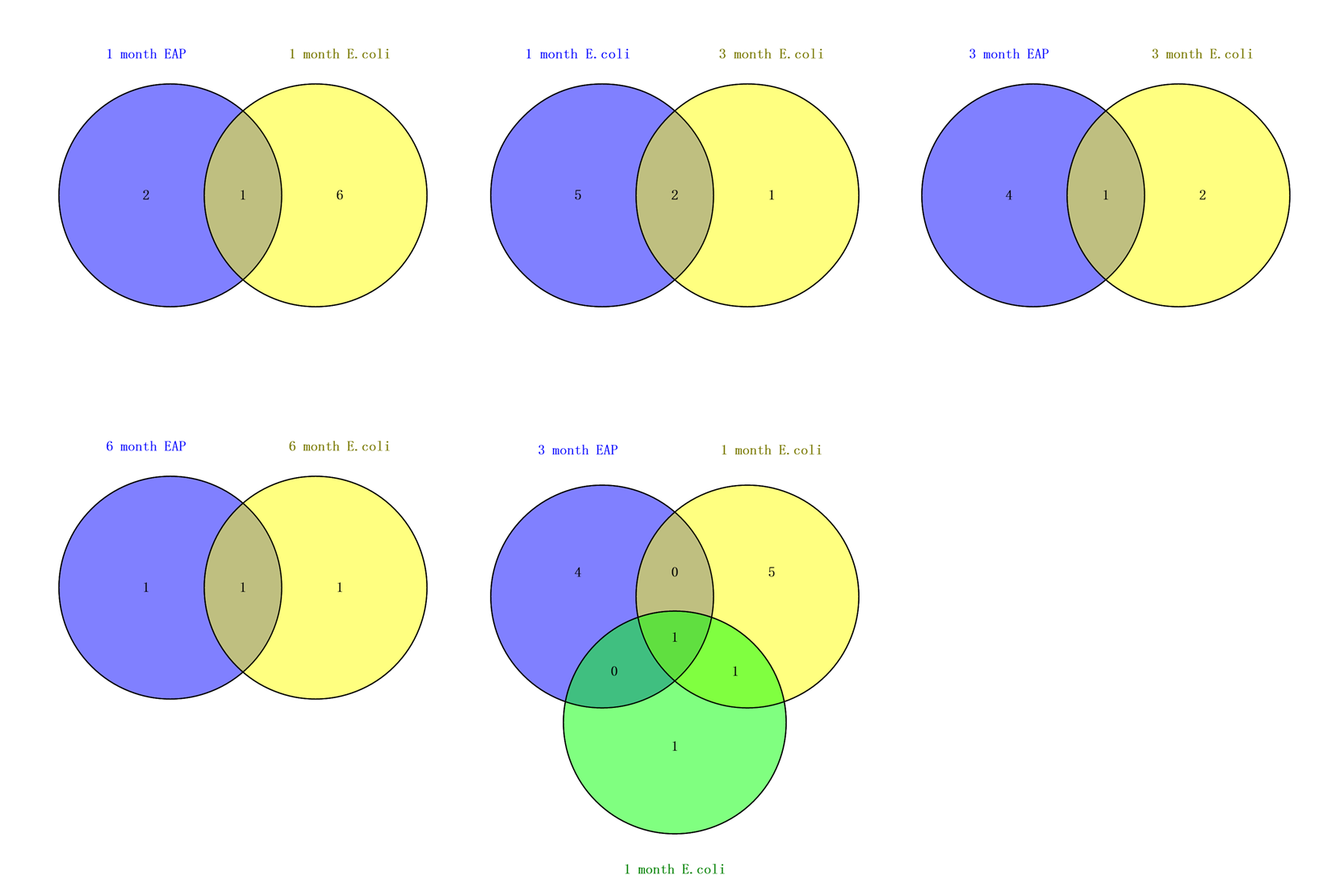

Supplement: Supplementary file 13 — Additional file 13: Figure S9. Venn diagram showing the overlap mutation gene among naïve, CFA, EPA, PBS, and E. coli groups at different time points of 1, 3, and 6 months. [file 40659_2019_237_MOESM13_ESM.tif]
