# Supplementary material for: Chronic prostatitis alters the prostatic microenvironment and accelerates preneoplastic lesions in C57BL/6 mice
Source: Biol Res. 2019 May 14;52:30. doi: 10.1186/s40659-019-0237-4 (PMC6518623; doi:10.1186/s40659-019-0237-4)
Supplement: Supplementary file 8 — Additional file 8: Table S1. SNP and index exclusion criteria. [file 40659_2019_237_MOESM8_ESM.docx]

**Table S1. SNP and index exclusion criteria.**

| SNP exclusion criteria | Indel exclusion criteria |
| --- | --- |
| Quality by depth(QD) < 2.0 | Quality by depth(QD) < 2.0 |
| RMS mapping quality(MQ) < 40.0 | RMS mapping quality(MQ) < 40.0 |
| Coverage(DP) <= 6 | Coverage(DP) <= 6 |
| Z-Score from Wilcoxon rank sum test of alternative versus reference read mapping qualities(MQRankSum) < -12.5 | Z-Score from Wilcoxon rank sum test of alternative versus reference read mapping qualities(MQRankSum) < -12.5 |

Variants meeting one or more of these criteria were excluded from further analysis of genomic diversity
