# Supplementary material for: Chronic prostatitis alters the prostatic microenvironment and accelerates preneoplastic lesions in C57BL/6 mice
Source: Biol Res. 2019 May 14;52:30. doi: 10.1186/s40659-019-0237-4 (PMC6518623; doi:10.1186/s40659-019-0237-4)
Supplement: Supplementary file 9 — Additional file 9: Table S2. Gene exon variants in CFA, EPA, E. coli, PBS and naive group at 1, 3, 6 month after injection or infection. [file 40659_2019_237_MOESM9_ESM.docx]

**Table S2. Gene exon variants in CFA, EPA, E.coli, PBS and naive group at 1, 3, 6 month after injection or infection.**

| **group** | **Sample ID** | **Variant (sum)** | **Frameshift mutation** | **Non-frameshift mutation** | **Synonymous mutaion** | **Non-synonymous mutation** | **Stop gain** | **Stop loss** |
| --- | --- | --- | --- | --- | --- | --- | --- | --- |
| E.coli and PBS group | | | | | | | | |
| 1 month E.coli | A26 | 1376 | 22 | 21 | 473 | 828 | 31 |  |
| 1 month E.coli | A72 | 1381 | 24 | 19 | 480 | 828 | 29 |  |
| 1 month E.coli | A75 | 1376 | 22 | 21 | 492 | 809 | 31 |  |
| 1 month PBS | A55 | 1360 | 21 | 25 | 465 | 820 | 28 |  |
| 1 month PBS | A96 | 3726 | 35 | 39 | 1779 | 1828 | 44 | 2 |
| 1 month PBS | A95 | 1677 | 22 | 20 | 706 | 896 | 32 | 1 |
| 3 month E.coli | A91 | 3305 | 32 | 36 | 1465 | 1729 | 42 |  |
| 3 month E.coli | A78 | 1398 | 21 | 20 | 491 | 835 | 30 |  |
| 3 month E.coli | A82 | 1460 | 24 | 20 | 535 | 850 | 30 |  |
| 3 month PBS | A28 | 1482 | 23 | 20 | 557 | 852 | 29 |  |
| 3 month PBS | A49 | 1375 | 20 | 20 | 476 | 829 | 29 |  |
| 3 month PBS | A64 | 2026 | 23 | 22 | 990 | 960 | 30 |  |
| 6 month E.coli | A1 | 1527 | 24 | 19 | 576 | 880 | 27 |  |
| 6 month E.coli | A53 | 1531 | 22 | 24 | 556 | 896 | 32 |  |
| 6 month E.coli | A39 | 1413 | 23 | 18 | 497 | 845 | 29 |  |
| 6 month PBS | A13 | 1992 | 22 | 21 | 947 | 975 | 26 |  |
| 6 month PBS | 14 |  |  |  |  |  |  |  |
| 6 month PBS | A84 | 2110 | 22 | 18 | 1054 | 983 | 32 |  |
| **EAP and CFA group** | | | | | | | | |
| 1 month EAP | S5 | 1616 | 26 | 20 | 608 | 930 | 31 |  |
| 1 month EAP | S25 | 1497 | 24 | 21 | 554 | 866 | 31 | 1 |
| 1 month EAP | S31 | 1414 | 22 | 19 | 496 | 846 | 30 |  |
| 1 month CFA | S50 | 1412 | 23 | 21 | 510 | 827 | 30 |  |
| 1 month CFA | S54 | 1414 | 22 | 21 | 478 | 863 | 29 |  |
| 1 month CFA | S71 | 1490 | 18 | 20 | 556 | 867 | 28 |  |
| 3 month EAP | S44 | 1816 | 25 | 20 | 857 | 882 | 31 |  |
| 3 month EAP | S51 | 1418 | 24 | 21 | 489 | 854 | 29 |  |
| 3 month EAP | B3 | 3437 | 31 | 36 | 1555 | 1774 | 40 | 1 |
| 3 month CFA | S17 | 1385 | 21 | 19 | 480 | 835 | 29 |  |
| 3 month CFA | S37 | 2253 | 24 | 21 | 1111 | 1067 | 29 |  |
| 3 month CFA | S48 | 1382 | 20 | 21 | 472 | 837 | 31 |  |
| 6 month EAP | S6 | 1599 | 29 | 19 | 595 | 923 | 32 |  |
| 6 month EAP | S26 | 1372 | 25 | 19 | 475 | 825 | 27 |  |
| 6 month EAP | S24 | 1386 | 22 | 20 | 477 | 835 | 31 |  |
| 6 month CFA | S14 | 1406 | 23 | 21 | 499 | 832 | 30 |  |
| 6 month CFA | S21 | 1450 | 21 | 24 | 551 | 825 | 28 |  |
| 6 month CFA | S36 | 1425 | 25 | 21 | 492 | 855 | 31 |  |
| **Naïve group** | | | | | | | | |
| 1 month naïve | S18 | 1891 | 23 | 22 | 881 | 934 | 30 |  |
| 1 month naïve | S81 | 3460 | 30 | 35 | 1554 | 1799 | 41 |  |
| 1 month naïve | K1 | 1424 | 24 | 21 | 488 | 859 | 31 |  |
| 3 month naïve | W1 | 1463 | 24 | 19 | 506 | 883 | 30 |  |
| 3 month naïve | W3 | 1485 | 22 | 21 | 535 | 876 | 30 |  |
| 3 month naïve | W8 | 1467 | 28 | 22 | 495 | 887 | 34 |  |
| 6 month naïve | B5-1 | 1369 | 21 | 22 | 483 | 814 | 28 |  |
| 6 month naïve | S80 | 3419 | 29 | 38 | 1530 | 1780 | 41 |  |
| 6 month naïve | B5-2 | 3118 | 30 | 31 | 1354 | 1660 | 42 |  |
